# *Cryptosporidium* spp. in Argentina: epidemiology and research advances in human, animal, and environmental settings during the 21st century

**DOI:** 10.3389/fmicb.2025.1592564

**Published:** 2025-05-27

**Authors:** Maria Romina Rivero, Claudina Vissio, Constanza Feliziani, Carlos De Angelo, Maria Carolina Touz, Karina Tiranti, Joaquín Andrés Lombardelli, Florencia Judith Duartez, Lumila Curletto

**Affiliations:** ^1^Instituto de Desarrollo Agroindustrial y de la Salud (IDAS-CONICET), Consejo Nacional de Investigaciones Científicas y Técnicas, CONICET, Universidad Nacional de Rio Cuarto, Rio Cuarto, Argentina; ^2^Instituto de Investigación Médica Mercedes y Martín Ferreyra (INIMEC), Consejo Nacional de Investigaciones Científicas y Técnicas (CONICET), Universidad Nacional de Córdoba, Córdoba, Argentina; ^3^Instituto de Ciencias de la Tierra, Biodiversidad y Ambiente (ICBIA – UNRC-CONICET), Consejo Nacional de Investigaciones Científicas y Técnicas, CONICET, Universidad Nacional de Rio Cuarto, Rio Cuarto, Argentina; ^4^Departamento de Patología Animal, Facultad de Agronomía y Veterinaria, Universidad Nacional de Rio Cuarto, Rio Cuarto, Argentina

**Keywords:** *Cryptosporidium*, prevalence, zoonotic potential, diagnostic techniques, risk factors, one-health, regional variations, human-animal and environmental health

## Abstract

**Introduction:**

*Cryptosporidium* spp. is an intestinal protozoan causing cryptosporidiosis, a diarrheal disease affecting humans and animals, with zoonotic potential. In immunocompromised individuals, infections can be severe or fatal. It is a major waterborne parasite and a leading cause of neonatal diarrhea in calves. This study systematically reviews *Cryptosporidium* spp. research in Argentina during the 21st century, highlighting its epidemiological significance and research gaps.

**Methods:**

A systematic review following PRISMA guidelines was conducted using LILACS, PubMed, Scopus, and SciELO Argentina. Eligible studies (2001–2024) included human (community and hospital-based), animal (domestic, wild, and captive), and environmental (water, soil and vegetable) surveys. The review analyzed epidemiology, diagnosis, treatment, genetic diversity, distribution, and risk factors.

**Results:**

Of 277 articles reviewed, 66 met eligibility criteria. *Cryptosporidium* spp. was detected in 17 of Argentina’s 23 provinces, mainly in the Pampean region. Five species were identified (*C. hominis, C. parvum, C. suis, C. scrofarum,* and *C. varanii*), though genetic diversity studies remain limited. Human cryptosporidiosis primarily affects immunocompromised individuals (HIV/AIDS, transplant recipients, hematologic cancer patients). The parasite was found in feces, duodenal biopsies, blood, sputum, and cerebrospinal fluid, with complications such as cholangiopathy and pulmonary cryptosporidiosis. Infections with *C. hominis* and *C. parvum* (including co-infections) were observed, with multiple subtypes documented. In animals, *C. parvum* was prevalent in Pampean calves, while *C. suis* and *C. scrofarum* were found in domestic pigs. Wildlife, including non-human primates and coypu, also tested positive. *Cryptosporidium* was detected in recreational and drinking water samples. No *Cryptosporidium* spp. oocysts were detected in soil. Risk factors included socio-economic conditions and animal management practices.

**Conclusion:**

*Cryptosporidium* spp. is widely distributed in Argentina, yet eco-epidemiological transmission factors remain poorly understood, hindering control strategies. Limited research on genetic diversity and distribution highlights the need for further studies, particularly in vulnerable populations and areas of close human-animal interaction, such as productive systems. The presence of *Cryptosporidium* spp. in water underscores the importance of improving public health policies and water treatment standards. From a One Health perspective, these findings emphasize the need for enhanced epidemiological surveillance and research to strengthen prevention and control in Argentina.

## 1 Introduction

Enteric protozoal infections are a recognized global concern for both animal and public health (Fletcher et al., 2012; Kotloff et al., 2019). *Cryptosporidium* spp. is an intestinal protozoan parasite from the Apicomplexa phylum that causes cryptosporidiosis, which is considered a leading cause of diarrheal disease worldwide (Thompson et al., 2005). More than 40 species of *Cryptosporidium* have been identified using advanced molecular techniques (Feng et al., 2018). This genus has a wide range of hosts, including humans, livestock, companion animals, wildlife, birds, reptiles, and fish (Ryan et al., 2014). *Cryptosporidium*’s complete genome has highlighted the uniqueness of this organism in terms of its parasitic lifestyle and evolutionary biology (Abrahamsen et al., 2004; Xu et al., 2004). It is an obligate intracellular parasite with a complex life cycle, primarily concentrated in the epithelial cells of the digestive tract of various host species. It causes gastrointestinal disease in its host species, ranging from asymptomatic or mild symptoms to severe illness in some instances. Infection results in the formation of oocysts, which are subsequently shed in the feces of affected hosts (Tzipori et al., 2002; Smith et al., 2005; Xiao and Cama, 2018).

The oocysts of *Cryptosporidium* spp. are highly resistant to environmental stressors, including chlorine treatments commonly used to disinfect drinking water. As a result, *Cryptosporidium* spp. are a ubiquitous water contaminant that serves as efficient vehicles for transmission, making them major water-and food-borne pathogens (Korich et al., 1990; King and Monis, 2007; Yang et al., 2012; Rosado-García et al., 2017). While the infection has significant gastrointestinal impacts, extra-intestinal and long-term consequences of *Cryptosporidium* spp. infections in animals and humans have also been described (Fleta et al., 1995; Iglói et al., 2018; Ungar, 2018; Chaudhari et al., 2021). In 2004, the World Health Organization included *Cryptosporidium* spp. in its “Neglected Disease Initiative” due to their considerable public health and socioeconomic impact (Savioli et al., 2006). In recent decades, the impact on both human and veterinary health has been increasingly recognized internationally, and its zoonotic potential is continuously updated (Ryan et al., 2021). Due to the unavailability of safe drugs or vaccines, *Cryptosporidium* spp. have emerged as a significant public health concern (Dhal et al., 2022). Additionally, due to the environmental persistence of oocysts, *Cryptosporidium* spp. pose significant public health challenges. This underscores the importance of adopting a One Health approach (Pinto and Vinayak, 2021). Indeed, an increasing number of researchers advocate the implementation of this approach to effectively address cryptosporidiosis, highlighting the relevance of collaborative, interdisciplinary efforts in line with the One Health framework (Innes et al., 2020). Numerous international reviews have characterized the eco-epidemiological aspects of this pathogen worldwide (Ryan and Power, 2012; Mahmoudi et al., 2017; Robertson et al., 2020) highlighting its complex transmission dynamics and the necessity for integrated control measures.

In developing countries, cryptosporidiosis is a major cause of diarrhea in young children, particularly in low-and middle-income countries (Korpe and Bartelt, 2015; Rosado-García et al., 2017; Squire et al., 2017; Robertson et al., 2020; Yang et al., 2021; Jann et al., 2022). It has been recognized as one of the most important causes of moderate to severe diarrhea and diarrhea-related mortality in children under 2 years of age in multiple recent studies. Significant outbreaks in humans due to contaminated water have also been reported. The pathogenic role of *Cryptosporidium* spp. in bovine hosts—manifesting primarily as diarrheal disease in both beef and dairy cattle—has been extensively documented (Santin, 2020). More recently, there have been reports of *Cryptosporidium* spp. infections in a wide variety of wild animals (Zahedi et al., 2016; Hatam-Nahavandi et al., 2019), as well as updates on its prevalence in farm animals such as pigs and poultry (Nakashima et al., 2022).

Despite extensive global studies on *Cryptosporidium* spp., regional data, particularly from Latin America and Argentina, remain scarce and less comprehensively documented. Argentina, the second largest country in Latin America after Brazil, spans an area of 2,791,810 km^2^ and is located between 22° and 55° south latitude and 53° and 74° west longitude. This vast country has diverse climatic conditions from north to south and east to west. National compilations on the prevalence and distribution of intestinal parasites show different profiles depending on climatic regions and socioeconomic conditions (Ministerio de Salud y Ambiente de Argentina, 2004; Juarez and Rajal, 2013; Socias et al., 2014; Rivero et al., 2020). The central region (the Pampas) is the wealthiest and concentrates the main agricultural and livestock activities, while the northern region is less developed, with high levels of poverty (INDEC, 2016). Multiple studies have reported the presence of intestinal protozoan parasites in humans, animals, and environmental sources in Argentina. However, a comprehensive synthesis at the national level on the prevalence, geographical distribution, and risk factors associated with *Cryptosporidium* spp. infections is still lacking. Moreover, cryptosporidiosis is not a notifiable disease in the country, further complicating surveillance efforts.

This review aims to comprehensively analyze *Cryptosporidium* spp. in Argentina during the 21st century, integrating epidemiological data from human, animal, and environmental studies. Additionally, it examines diagnostic advancements, treatment strategies, and prevention measures. By identifying key research gaps and mapping the pathogen’s distribution, this study seeks to enhance the understanding of cryptosporidiosis in Argentina and inform future public health policies within a One Health framework.

## 2 Methods

### 2.1 Systematic search strategy and eligibility criteria

A systematic literature review was conducted in accordance with the PRISMA-P (Preferred Reporting Items for Systematic Review and Meta-Analysis Protocols) guidelines (Moher et al., 2015). The search was performed across four electronic databases: LILACS, PubMed, Scopus, and Argentina SciELO, covering the period from January 2001 to December 2024. The search strategy used the keywords: “*Cryptosporidium*” AND “Argentina” AND “Prevalence” AND “Risk factors,” with syntax adjusted to meet the requirements of each database. In PubMed, filters were applied to include studies involving humans and animals, restrict language to English, Spanish, or Portuguese, and limit publication type to journal articles. In Scopus, results were refined by subject areas (Agricultural and Biological Sciences, Medicine, Environmental Science) and limited to peer-reviewed publications. Searches in LILACS and SciELO were conducted using DeCS/MeSH-equivalent terms, focusing on original articles, case reports, and communications published in indexed scientific journals. Additionally, a manual review of reference lists from selected articles was performed to identify additional relevant publications.

**Figure 1:**
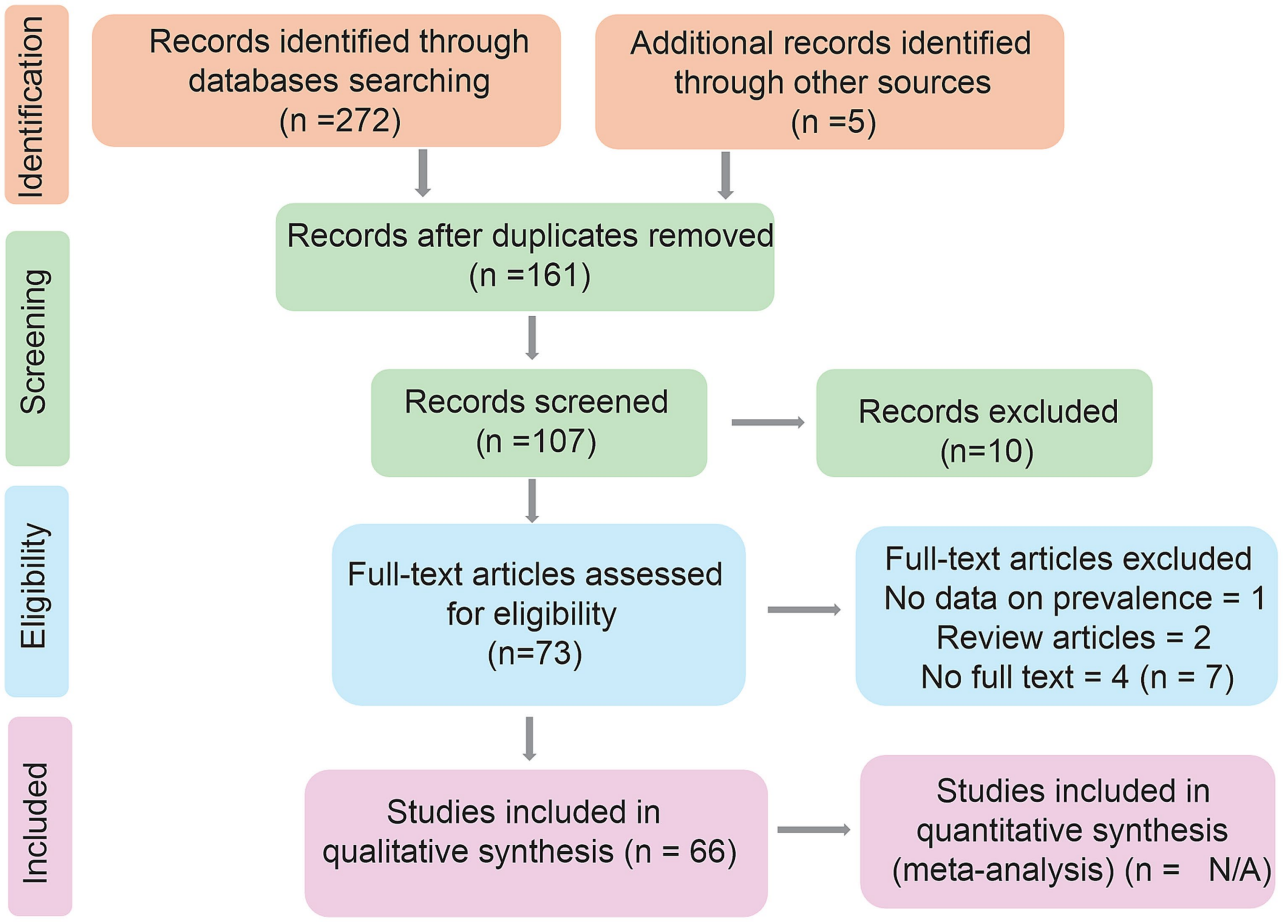
PRISMA flowchart filled with final data. The strategy used for study inclusion in the final analysis is summarized (Moher et al., 2015).

Studies were included if they were original research articles, brief communications, academic theses, or case reports published in peer-reviewed journals with an ISSN, and written in English, Spanish, or Portuguese. Eligible studies reported prevalence, risk factors, detection techniques, molecular characterization, or prevention and control strategies related to *Cryptosporidium* spp. in humans, animals, or environmental samples in Argentina. Literature reviews, systematic reviews, and conference abstracts without full peer-reviewed publications were excluded. Also excluded were studies that addressed intestinal parasitoses in general without specifying data on *Cryptosporidium* spp. as well as duplicated studies or reports presenting the same dataset in multiple sources. These criteria were applied during both the title and abstract screening and full-text review phases by two independent reviewers, with disagreements resolved by consensus (Figure 1).

### 2.2 Study selection and data extraction

Studies were categorized based on human, animal, and environmental settings. Those studies that included combined sampling and/or different locations were analyzed in all the applicable geographic locations and epidemiological settings. Prevalence studies in humans and animals included those with prevalence data or sufficient information for their calculation, while environmental surveys considered occurrence data. Data was organized in MS Excel, and coding was performed to summarize information on the study location, study design, population, sampling framework, methodology for *Cryptosporidium* spp. detection and the reported treatment scheme.

### 2.3 Mapping the epidemiological impact of *Cryptosporidium* spp

**Figure 2:**
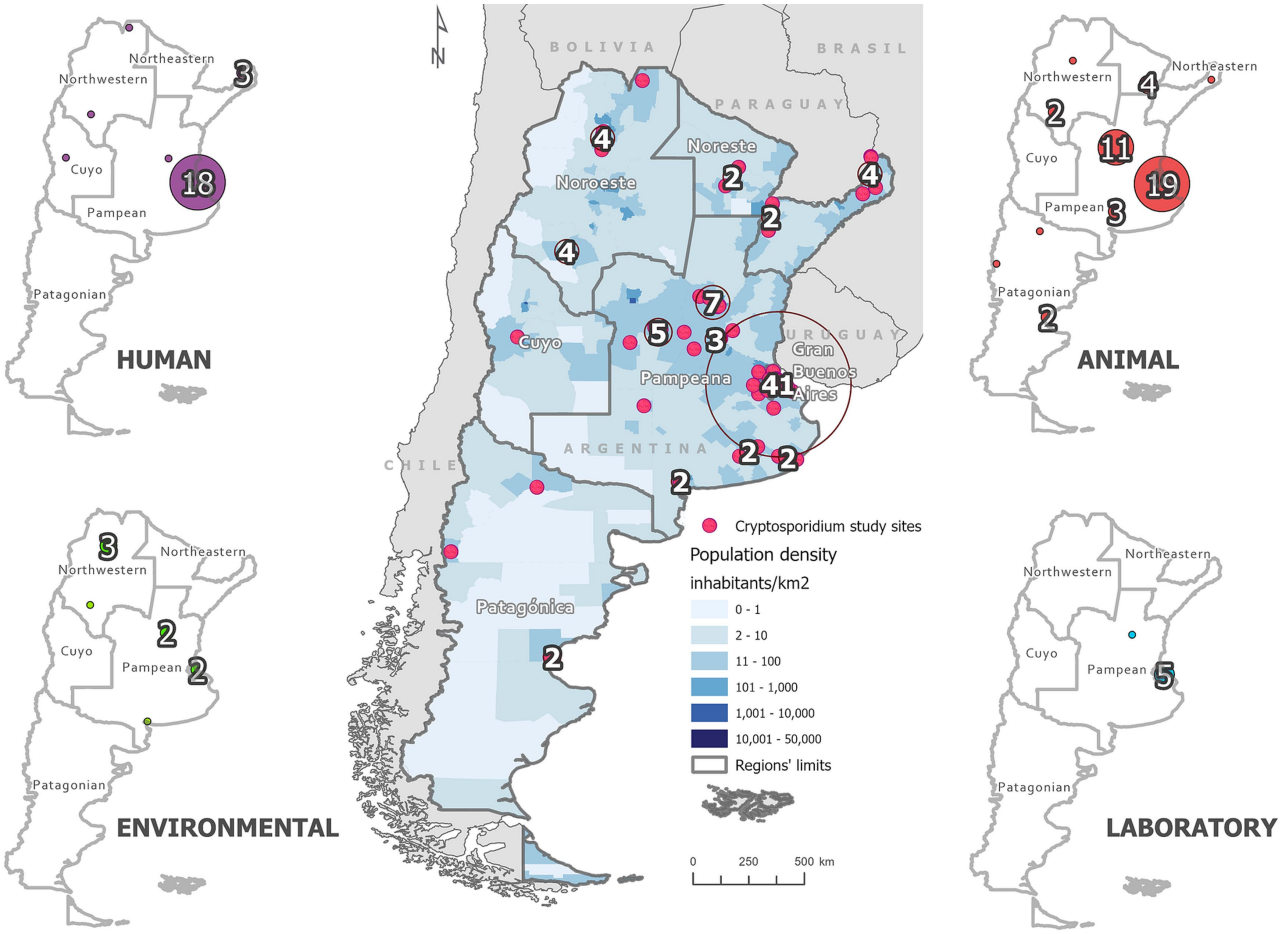
Geospatial distribution of eligible publications across Argentina, overlaid on population density based on the 2020 national census and regional divisions defined by the National Institute of Statistics and Censuses (INDEC, 2022). Red circles indicate the total number of studies published between 2001 and 2024 in each region. Side panels disaggregate the data by study type (human, animal, laboratory, and environmental), with markers identifying specific study locations and the number of studies per category. This spatial visualization highlights geographic disparities in research efforts, with a clear concentration of studies in more densely populated central regions and limited research activity in the northwestern and Patagonian areas. These patterns underscore existing research gaps and the need for broader territorial coverage in future investigations.

To visualize the distribution of *Cryptosporidium* spp. in Argentina during the 21st century, an epidemiological mapping was performed based on the seven regions defined by the National Institute of Statistics and Censuses of Argentina (INDEC): Northeast, Northwest, Cuyo, Pampean, Great Buenos Aires (we include the studies conducted in the Autonomous City of Buenos Aires, CABA), Patagonian and Antarctic region. This regionalization considers geographic, population, economic, and climatic data, as well as political-territorial-administrative organization (Figure 2). Based on this, the data obtained from selected publications on *Cryptosporidium* spp. detections reported in the analyzed period were included in a map of Argentina. Each selected study was geo-referenced and classified according to the sample setting (animal, environmental and human). When the locality was not specified, the one from the manuscript title was used. Laboratory research studies were also properly indicated. Regional distribution analysis was performed using ArcGIS Pro 3.4 (ESRI Inc.). *Cryptosporidium* spp. have not been found in the Argentine Antarctic Region; therefore, it is not included in the images to improve map visualization.

### 2.4 Risk factor analysis for *Cryptosporidium* presence

Risk factors and associated factors considered in the selected publications were classified and categorized according to each epidemiological setting (human, animal, and environmental). Association with others microbial hazards or contamination indicators were also summarized by regions.

### 2.5 Statistical analysis

Statistical and graphical analyses were performed using R (R Development Core Team, 2020), with support from the Plotly package (Sievert, 2020).

## 3 Results

### 3.1 Systematic review: research settings and geographical distribution analysis

**Figure 3:**
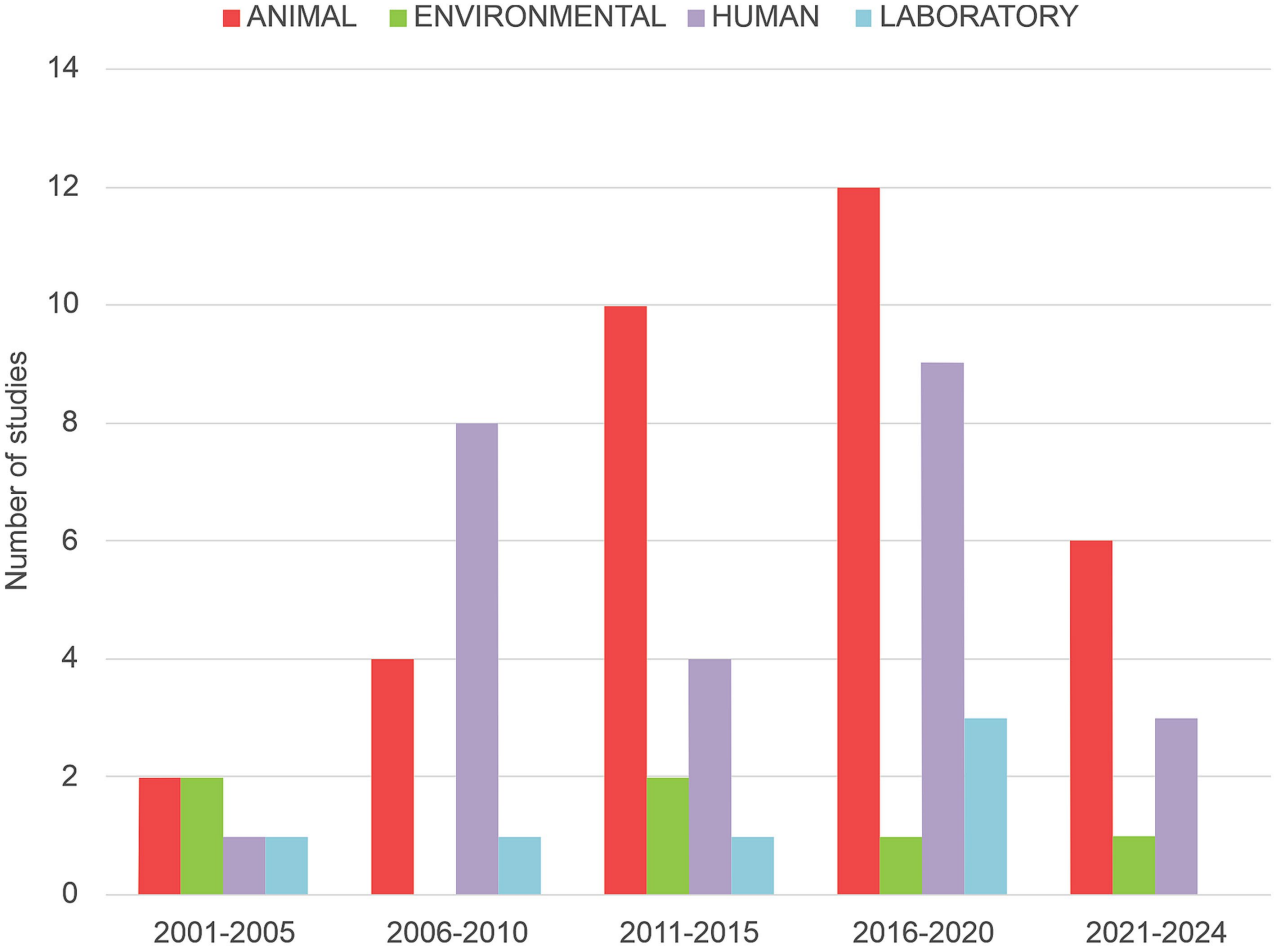
Temporal distribution of publications on *Cryptosporidium* spp. in Argentina from 2001 to 2024, categorized by study setting (human, animal, laboratory, environmental) and grouped into five-year intervals. This timeline illustrates trends in research output over the 21st century, beginning with a low number of studies and showing a marked increase starting in the 2011–2015 period, with the sharpest rise observed from 2016 to 2020. The chart also highlights differences in focus by setting, with human and animal studies being more consistently represented, while environmental and laboratory studies appear more sporadically. These dynamics reflect the growing scientific interest in *Cryptosporidium* spp., influenced by advances in detection methods and increased awareness of its relevance in One Health frameworks.

Two hundred seventy-seven studies were initially identified following PRISMA guidelines (Moher et al., 2015). Of these, 66 studies published between 2001 and 2024 met the inclusion criteria and were included in the subsequent analysis (Figure 2). Some studies combined samples across different epidemiological settings, resulting in a total of 82 reports included in the subsequent analysis. The analysis of research settings revealed that 51.2% of observational studies were conducted in animal settings, followed by 30.5% in humans, 11.0% in environmental, and 7.3% in laboratory contexts. *Cryptosporidium* spp. have been reported in 17 of Argentina’s 23 provinces, encompassing all continental regions. Notably, publications on intestinal parasitoses involving *Cryptosporidium* spp. increased significantly between 2011 and 2020, with the sharpest rise observed from 2016 to 2020 (Figure 3). Geographically, the Pampean region, particularly Buenos Aires Province, had the highest number of studies, followed by the Greater Buenos Aires (GBA) region, while the Patagonian and Cuyo regions reported the fewest (Figure 2; Table 1). Among the northern provinces, Misiones (Northeast region), and Salta and La Rioja (Northwest region) had the highest number of surveys (Figure 2; Table 1). The contribution of studies from the provinces in the Patagonian region is similar, while in the Cuyo region, only Mendoza province reported a single study on *Cryptosporidium* spp. during the study period (Figure 2; Table 1). All laboratory studies were conducted in the Pampean region. They were developed at National Universities, the National Council for Scientific and Technical Research (CONICET), or National Institute of Agricultural Technology (INTA) in Buenos Aires and Santa Fe provinces (See section Laboratory Research).

**Table 1:** Distribution of studies by region, province, and sphere in Argentina (2001–2024), including number of sites surveyed, study proportion per region, year range, involvement of human, animal, and environmental spheres, and experimental approaches indicated in the laboratory column.

INDEC region (total sites surveyed)	Province	*N* (region %)	Year Range*	Sphere			
				Human	Animal	Environmental	Laboratory
Pampean (40)	Buenos Aires	24 (60.0)	2001–2023	5	14	2	3
	Santa Fe	9 (22.5)	2001–2020	1	5	2	1
	Córdoba	5 (12.5)	2011–2022		5		
	La Pampa	1 (2.5)	2020–2020		1		
	Entre Rios	1 (2.5)	2020–2020		1		
Great Bs As (21)	Caba	15 (71.4)	2008–2024	12	2		1
	Buenos Aires	6 (28.6)	2006–2024	1	3	1	1
Northwest (8)	Salta	5 (62.5)	2003–2021	1	1	3	
	La Rioja	3 (37.5)	2017–2017	1	1	1	
Northeast (8)	Misiones	4 (50.0)	2006–2020	3	1		
	Chaco	2 (25.0)	2011–2019		2		
	Corrientes	2 (25.0)	2011–2011		2		
Patagonian (4)	Chubut	2 (50.0)	2020–2020		2		
	Neuquen	1 (25.0)	2010–2010		1		
	Rio Negro	1 (25.0)	2021–2021		1		
Cuyo (1)	Mendoza	1 (100)	2007–2007	1			

*Publication year (not necessarily represents the year of the survey).

### 3.2 *Cryptosporidium* spp. in children and adults in Argentina

**Figure 4:**
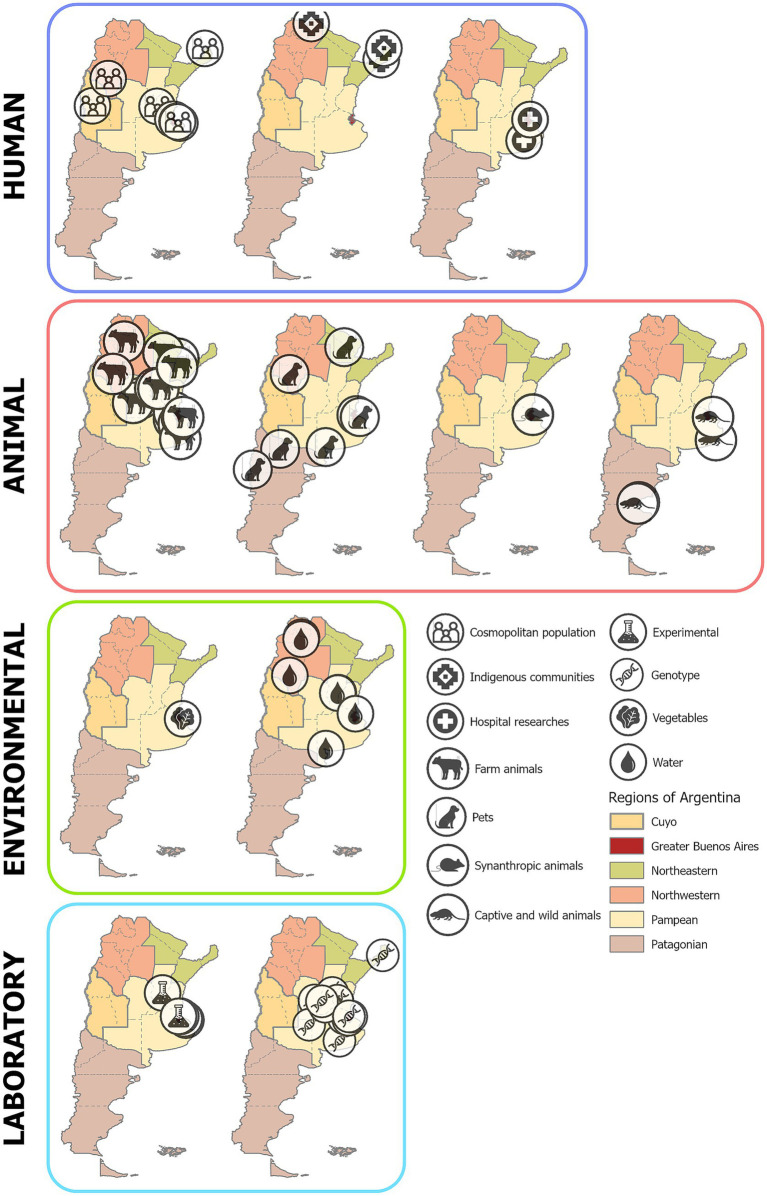
Map of studies on *Cryptosporidium* spp. prevalence by epidemiological setting. Human: from left to right – community-based, indigenous community surveys, hospital-based studies. Animals: from left to right – domestic animals, farm animals, pets, synanthropic animals, and wild animals. Laboratory: experimental studies and genotype investigations. Environmental: vegetable and water surveys. Original icons created by the authors and by Prayudawanto, ProSymbols, Athok, Evon, Salman Azzumardi, Sandra, Adrien Coquet, Kosong Tujuh, and Pramana from the Noun Project.

Epidemiological investigations throughout the 21st century have confirmed the presence of human cryptosporidiosis in at least 8 out of the 23 provinces of Argentina, based on both community-based and hospital-based studies, and genotyping research (Figure 4, Human; Table 1). A total of 12 community-based studies that analyzed human feces reported the occurrence of *Cryptosporidium*. Most of these studies focused on asymptomatic children, although a few included adults. Of the 4,435 asymptomatic individuals analyzed, an average of 2.9% (SD 4.44) tested positive for *Cryptosporidium*, with prevalence rates ranging from 0.1 to 7.3%. These rates varied according to age, nutritional status, socioeconomic conditions, province, and ethnicity, with the highest prevalence observed in children from peri-urban areas in the Great Buenos Aires and Pampean regions (Basualdo et al., 2007; Pezzani et al., 2009; Garbossa et al., 2013) and from indigenous communities in northern Argentina who were more socio-environmentally vulnerable (Taranto et al., 2003; Navone et al., 2006) (Table 2).

**Table 2:** Human cryptosporidiosis in Argentina during 2001–2024 from community-based studies.

Year*	Region of Argentina	Province	Study design	Prevalence	N	Population surveyed	Reference
2007	Cuyo	Mendoza	Community-based	1.3	221	Children from 1 to 14 years old	Salomon et al. (2007)
2013	Great Buenos Aires	Buenos Aires	Community-based	3.5	138	Children from 6 months to 13 years old	Garbossa et al. (2013)
2006	Northeast	Misiones	Indigenous villages	1.7	296	Individuals from 10 months to 82 years old	Navone et al. (2006)
2017	Northeast	Misiones	Community-based	0.6	483	Children from 0 to 15 years old	Rivero et al. (2017a, 2017b)
2018	Northeast	Misiones	Indigenous villages	0.7	303	Children from 0 to 15 years old	Rivero et al. (2018)
2007	Northwest	Salta	Indigenous villages	4.2	95	Individuals from 1 to 82 years old	Taranto et al. (2003)
2017	Northwest	La Rioja	Community-based	19.35^a^	93	Children from 0 to 15 years old	Cerezuela et al. (2017)
2007	Pampean	Buenos Aires	Community-based	6.90	504	Children and adults	Basualdo et al. (2007)
2009	Pampean	Buenos Aires	Community-based	7.3	522	Children and Adults	Pezzani et al. (2009)
2021	Pampean	Buenos Aires	Community-based	0.1	1,411	Children under 14 years old	Cociancic et al. (2018)
2020	Pampean	Buenos Aires	Community-based	2.60	350	Individuals from 1 to 65 years old	Falcone et al. (2020)
2011	Pampean	Santa Fe	Community-based	2.7	112	Children from 4 months to 16 years old	Indelman et al. (2011)

*Publication year (not necessarily represents the year of the survey).

a The prevalence reported in this study was excluded because the national-level analysis only includes data from asymptomatic children.

Regarding regional distribution, *Cryptosporidium* spp. was reported in humans from 5 out of the 7 INDEC regions in Argentina (Table 2) Most studies were cross-sectional observational studies on the prevalence of intestinal parasites in general, rather than focusing specifically on *Cryptosporidium* spp. Individuals for copro-parasitological analysis were primarily recruited through schools and/or local public healthcare centers, which also served as sample collection points. In the Northeast region, samples were gathered during house-to-house visits, with the support of indigenous translators and community health workers (Rivero et al., 2017a; Rivero et al., 2018).

Six hospital-based studies conducted in the Pampa region reported the presence of *Cryptosporidium* spp. (Table 3), with an additional three studies evaluating the genetic diversity of the parasite (Table 4).

**Table 3:** Human cryptosporidiosis in Argentina during 2001–2024 from hospital-based studies.

Year*	Region of Argentina	Province	Study Design	Hospital	Population surveyed	Reference
2024	Pampean region	Buenos Aires	Hospital-based	Pediatric Hospital “Prof. Dr. Juan P. Garrahan”	Children with diseases associated with immunosuppression (solid organ transplant, had a hematologic neoplasm, or primary immunodeficiency).	Dumas Marucci et al. (2024)
2010	Pampean region	Buenos Aires	Hospital-based	General Municipal Hospital of Acute Diseases “Dr. José María Penna” and Infectious Diseases Hospital “Dr. Francisco Javier Muñiz”	HIV seropositive adult patients	Velásquez et al. (2010)
2008	Pampean region	Buenos Aires	Hospital-based	General Children’s Hospital “Dr. Pedro de Elizalde”	HIV seropositive children patients	Barboni et al. (2008)
2019	Pampean region	Buenos Aires	Hospital-based	Children’s Hospital “Doctor Debilio Blanco Villegas”	Children with gastric disorders	Molina et al. (2019)
2008	pampean region	Buenos Aires	Hospital-based	Infectious Diseases Hospital “Dr. Francisco Javier Muñiz.”	HIV seropositive adult patients	Corti et al. (2008)
2017	pampean region	Buenos Aires	Hospital-based	Infectious Diseases Hospital “Dr. Francisco Javier Muñiz”	Individuals with gastric disorders from 1 to 86 years old	Astudillo and Bava (2017)

*Publication year (not necessarily represents the year of the survey).

**Table 4:** Genetic analysis of cryptosporidiosis in human patients.

Year*	Region of Argentina	Province	Study design/sample	*Cryptosporidium* spp.	*Subgenotype*	Population surveyed	Reference
2012	Pampean	Buenos Aires	nPCR and RFLP/Gastrointestinal biopsy	*C. hominis, C. parvum*	ND^a^	HIV seropositive adult patients	Velásquez et al. (2012)
2018	Pampean	Buenos Aires	nested PCR-RFLP of the 18S rDNA, real-time PCR, and gp60 subtyping/Distal duodenum biopsies and stool samples	*C. hominis*	IeA11G3T3	HIV seropositive adult patients	Velásquez et al. (2018)
2016	Pampean	Buenos Aires	18S rRNA gene and RFLP analysis. Subtyped by DNA sequencing of the gp60 gene amplified by a nPCR/ Duodenum biopsies and stool samples	*C. hominis, C. parvum* and mixed infection	IbA10G2, IaA10G1R4, IaA11G1R4, IeA11G3T3, Ia and IIa.	HIV seropositive adult patients	Peralta et al. (2016)

*Publication year (not necessarily the year of the survey).

a ND: Not determined.

### 3.3 *Cryptosporidium* spp. diagnosis and treatment in humans

In Argentina, *Cryptosporidium* spp. have been detected in a variety of biological samples, including feces, duodenal biopsies, blood, sputum, aspirated bronchial material, and cerebrospinal fluid. The most employed diagnostic methods for detecting *Cryptosporidium* spp. involved concentration techniques applied to preserved stool samples, which were collected serially. These samples were then analyzed using conventional microscopic examination and staining methods (Velásquez et al., 2010; Peralta et al., 2016; Rivero et al., 2017a). Less frequently, detection was achieved with single stool samples, FLOTAC, or polymerase chain reaction (PCR) (Molina et al., 2007; Cimino et al., 2015). Unfortunately, many studies have not reported treatment protocols for *Cryptosporidium* spp. infections. In hospital-based studies, although the authors primarily focus on describing the underlying diseases of patients and their intestinal symptoms, only one study has specifically addressed the treatment of cryptosporidiosis. Azithromycin was identified as the only drug used in combination with highly active antiretroviral therapy (HAART) for HIV-positive patients. This strategy restores immune function and reduces both acute diarrheal episodes and hydroelectrolytic complications in individuals co-infected with *C. parvum* (Barboni et al., 2008). However, it is known that some patients do not respond to this treatment. The rising number of patients with different causes of immunosuppression has underscored the need to find effective treatment for *Cryptosporidium* spp. infections.

### 3.4 *Cryptosporidium* spp. in animals

*Cryptosporidium* spp. have been reported in 56 investigations across 44 studies, involving a wide range of domestic, farm, and wild animals (Figure 4, Animal; Table 5). These findings highlight the parasite’s broad host range and its potential for transmission across different environments.

**Table 5:** *Cryptosporidium* species and subtypes identified in animals in Argentina.

Year*	Argentina region	Province	Study design	*Cryptosporidium* spp.	*Subtypes*	Sampling sites	Reference
2013	Pampean	Buenos Aires	18S rRNA gene and RFLP analysis. GP60 gene subtyping	*C. parvum*	IIaA17G1R1, IIaA20G1R1, IIaA21G1R1, IIaA22G1R1 and IIaA23G1R1	Dairy farm	Tomazic et al. (2013)
2013	Pampean	Santa Fe	18S rRNA gene and RFLP analysis. GP60 gene subtyping	*C. parvum*	IIaA20G1R1, IIaA21G1R1, IIaA22G1R1 and IIaA23G1R1	Dairy farm	Tomazic et al. (2013)
2013	Pampean	Córdoba	18S rRNA gene and RFLP analysis. GP60 gene subtyping	*C. parvum*	IIaA18G1R1, IIaA20G1R1 and IIaA21G1R1	Dairy farms	Tomazic et al. (2013)
2019	Pampean	Córdoba	18S rRNA gene and RFLP analysis. GP60 gene subtyping	*C. parvum*	IIaA18G1R1, IIaA20G1R1, IIaA21G1R1, IIaA22G1R1, and IIaA24G1R1	Dairy farms	Lombardelli et al. (2019)
2014	Pampean	Buenos Aires	18S rRNA gene and RFLP analysis. GP60 gene subtyping	*C. parvum*	IIaA21G1R1, IIaA16G1R, IIaA18G1R1, IIaA19G1R1, IIaA20G1R, IIaA21G1R, IIaA22G1R1 and IIaA23G1R1.	Dairy farms	Del Coco et al. (2014)
2020	Pampean	La Pampa	18S rRNA gene and RFLP analysis. Species-specific nested PCR.	*C. suis, C. scrofarum*	ND^a^	Pig farms under the intensive breeding system	De Felice et al. (2020)
2020	Pampean	Buenos Aires	18S rRNA gene and RFLP analysis. Species-specific nested PCR.	*C. suis, C. scrofarum*	ND^a^	Pig farms under the intensive breeding system	De Felice et al. (2020)
2020	Pampean	Entre Ríos	18S rRNA gene and RFLP analysis. Species-specific nested PCR.	*C. suis,*	ND^a^	Pig farms under the intensive breeding system	De Felice et al. (2020)
2020	Northeast	Misiones	18S rRNA gene and RFLP analysis. Species-specific nested PCR.	*C. scrofarum*	ND^a^	Pig farms under the intensive breeding system	De Felice et al. (2020)
2020	Northeast	Santa Fe	18S rRNA gene and RFLP analysis. Species-specific nested PCR.	*C. scrofarum*	ND^a^	Pig farms under the intensive breeding system	De Felice et al. (2020)
2020	Northeast	Córdoba	18S rRNA gene and RFLP analysis. Species-specific nested PCR.	*C. suis, C. scrofarum*	ND^a^	Pig farms under the intensive breeding system	De Felice et al. (2020)
2016	Pampean	Buenos Aires	18S rRNA gene and RFLP analysis. Species-specific nested PCR.	*C. varanii*	ND^a^	Geckos *(Eublepharis macularius)* from a breeder colony	Dellarupe et al. (2016)

a ND: not determined.

*Publication year (not necessarily represents the year of the survey).

#### 3.4.1 Farm animals

Throughout the 21st century, 19 studies focused on farm animals, including calves, pigs, sheep, goats, and horses. Among these, calves were the most studied, with 14 investigations. Research on calves remained consistent from 2001 to 2021. Most studies were conducted in the Pampean region, while the Northeast and Northwest regions each contributed only one study (Figure 4, Animal; Supplementary Table 1). In Argentina, few studies have assessed the prevalence of *Cryptosporidium* spp. in pigs, and its epidemiological and clinical characteristics. Pigs are highly susceptible to infection by various *Cryptosporidium* species, though the disease is usually subclinical. Only two studies have examined the epidemiological aspects of *Cryptosporidium* infection in intensive swine farms in the country (De Felice et al., 2020; Lovera et al., 2022) one of which also included genetic analysis. Both studies consistently reported that cryptosporidiosis is widely distributed in Argentina’s main pig husbandry region, with a low to moderate intra-farm infection rate. The distribution of *C. scrofarum* was higher than that of *C. suis*. Notably, both identified *Cryptosporidium* species have zoonotic potential. Recently, a study reported *Cryptosporidium* spp. co-occurrence with other protozoa parasites in pigs from family farms in the Northeast region highlights the necessity for molecular-level evaluations to detect potential zoonotic genotypes of the detected protozoa (Alegre et al., 2024), *Cryptosporidium* spp. has also been detected in horses, goats, and sheep. However, these studies are not population-based but are isolated investigations with small sample sizes.

#### 3.4.2 Pets and synanthropic animals

*Cryptosporidium* spp. have also been studied in companion animals, with eight investigations focusing on dogs (*Canis familiaris*). In the 21st century, only one study was published in the early 2000s, while the rest appeared after 2010. Most studies were conducted in Buenos Aires province, Neuquén (Patagonian region), and La Rioja (Northwest region) (Figure 4, Animal; Supplementary Table 1). Research on domestic cats (*Felis catus*) is notably scarce, with only one study reporting *Cryptosporidium* spp. in Pampean region (Figure 4, Animal; Supplementary Table 1). Exotic pets are also classified as synanthropic animals. In Buenos Aires Province, a study reported the presence of *Cryptosporidium* spp. in pet reptiles (Dellarupe et al., 2016).

*Cryptosporidium* spp. have also been detected in synanthropic rodents, with two studies focusing on these hosts (Figure 4, Animal; Supplementary Table 1). All studies were conducted by the same research group, which assessed the co-occurrence of *Cryptosporidium* spp. and *Giardia* spp. and the associated risk factors in brown rats (*Rattus norvegicus*) from Buenos Aires. The researchers emphasize that urban slums are particularly susceptible to supporting large populations of synanthropic rodents, which has important implications for the prevalence of *Cryptosporidium* spp.

#### 3.4.3 Captive animals and free-ranging wildlife

*Cryptosporidium* spp. have also been studied in captive mammals, with eight investigations involving species such as black howler monkey (*Alouatta caraya*), black spider monkeys (*Ateles* sp.), chimpanzees (*Pan troglodytes*), baboons (*Papio* sp.), tufted capuchins (*Sapajus apella*), and guinea pigs (*Cavia porcellus*). Argentina, with its diverse biogeographical regions and rich wildlife diversity (Arana et al., 2021), has reported *Cryptosporidium* spp. in several wild species. Two studies on wild animals were eligible for this research. One study detected the parasite in invertebrates, such as mussels (*Mytilus edulis*), while another reported its presence in coypu (*Myocastor coypus*) (Figure 4, Animal; Supplementary Table 1). These findings highlight the diverse range of host-pathogen interactions that *Cryptosporidium* spp. can encounter.

#### 3.4.4 *Cryptosporidium* spp. diagnosis and treatment in animals

In Argentina, cryptosporidiosis in calves is commonly managed with Halocur^®^ (halofuginone), a product manufactured in France and available only by prescription following national regulations and guidelines of the National Service of Food Safety and Quality (SENASA). A simplified dosing regimen is employed. In farms with a history of cryptosporidiosis, this treatment is used to control diarrhea caused by *Cryptosporidium parvum*, with therapy initiated within the first 24 to 48 h of life or within 24 h of symptoms onset to mitigate disease severity. Daily administration is critical; once the initial calf is treated, subsequent newborns should be systematically managed with the recommended dose and frequency if *C. parvum* symptoms persist in the herd.

In other livestock species, such as pigs, chickens, horses, and goats, cryptosporidiosis treatment is mainly supportive, focusing on rehydration and symptom management, as no specific effective treatment is available. Similarly, in canines and felines, treatment is also supportive and aimed at controlling diarrhea and dehydration, since no highly effective specific medication exists. Although azithromycin and toltrazuril—a triazine derivative with broad-spectrum coccidiocidal and antiprotozoal activity—have emerged as treatment options, professionals recommend supportive care due to the observed variability in response. In all cases, strict hygiene measures are essential to prevent reinfection.

#### 3.4.5 *Cryptosporidium* specie identification in animals

This parasite presents wide intraspecific genetic diversity (Ryan et al., 2014). During the first decade of the century, there are no reports of genetic studies on *Cryptosporidium* spp. These types of approaches were first reported in 2013 in calves (Tomazic et al., 2013) and more recently in pigs (De Felice et al., 2020) (Table 5). Subtyping in calves revealed the coexistence of various subtypes, some of which were identified for the first time in Argentina such as IIaA24G1R1 (Lombardelli et al., 2019). Several subtypes detected, such as IIaA17G1R1 and IIaA18G1R1, are strongly implicated in zoonotic transmission. Additionally, it is interesting that subtype IIA15G2R1, the overwhelmingly dominant subtype in cattle in most studies worldwide, has not been found in the country (Del Coco et al., 2014).

In pigs, *C. suis* and *C. scrofarum* were detected, the results suggest the species-specific nPCRs as useful tools to improve molecular *Cryptosporidium* infection diagnosis in pigs (De Felice et al., 2020). Interestingly, molecular identification of *C. varanii* was assessed in pet leopard geckos (*Eublepharis macularius*) from a breeder colony in Buenos Aires, representing the first description of this pathogen in pet reptiles in the country (Dellarupe et al., 2016).

### 3.5 *Cryptosporidium* spp. in environmental samples

*Cryptosporidium* spp. were detected in water samples in five studies conducted between 2001 and 2017 in the Northwest and Pampean regions (Figure 4). The earliest investigations were carried out in the Pampean region—specifically in Buenos Aires (Costamagna et al., 2005) and Santa Fe province (Abramovich et al., 2001)—while studies from the Northwest region (Salta and La Rioja provinces) were conducted during the 2010s (Poma et al., 2012; Cacciabue et al., 2014; Cerezuela et al., 2017). A recent study on microbiological hazards in Río de la Plata recreational areas detected *Cryptosporidium* spp. in both a heavily polluted and an apparently safe beach. Additionally, various viral and bacterial pathogens were identified, highlighting the need for comprehensive microbial monitoring to assess water quality more accurately and support health risk evaluations (Díaz et al., 2024). In general, these studies analyzed both recreational water sources (rivers, dams, streams, and public swimming pools) and drinking water (from dams, supply wells, and urban networks) to determine *Cryptosporidium* spp. levels and to assess their relationship with bacteriological and physicochemical water quality parameters, as well as with other waterborne parasites. However, these relationships vary depending on the sampling location, water source, and level of contamination. Water sampling and analysis were generally performed by American Public Health Association recommendations (APHA, 2005). Most published articles describe hollow fiber ultrafiltration of water, specific staining methods (i.e., Kinyoung or modified Zhiel Neelseen staining) and direct immunofluorescence (Merifluor^™^ Crypto) to oocysts detection. The results were reported as the presence of *Cryptosporidium* spp. oocysts, the number of oocysts per liter (or per 100 liters), or as the percentage of positive samples for *Cryptosporidium* spp. oocysts.

On the other hand, although numerous studies have been conducted to detect intestinal parasites in soil samples, none reported the presence of *Cryptosporidium* spp. oocysts. Furthermore, a recent study analyzed soil samples from greenhouses that produce leafy vegetables without positive oocyst detection. However, this work detected, *Cryptosporidium* spp. oocysts by processing leafy vegetable samples (lettuce) (Falcone et al., 2023). Leafy vegetables have overlapping, flexible leaves that can retain irrigation water longer than the soil, increasing the likelihood of detecting parasitic structures (Santomauro et al., 2024).

### 3.6 Risk factors for transmission

Reviewed data identifies several key risk factors associated with the acquisition of *Cryptosporidium* spp. infections across different settings (Table 6). In hospital-based studies, particularly involving pediatric and immunocompromised patients, infections were linked to immune suppression (including but not limited to HIV/AIDS). Community-based research highlighted associations with socio-demographic variables such as low household income, lack of access to improved water sources, and the presence of young children in the household, especially in regions with lower socio-economic development. In the livestock sector, common risk factors included poor hygiene in animal enclosures, high animal density, inadequate manure management, younger animal age—especially in calves—and environmental conditions like soil type and temperature. Environmental studies frequently identified the use of untreated surface water, inadequate sanitation infrastructure, and the presence of livestock near water sources as major contributors to contamination. However, few studies performed multivariate analyses, limiting the ability to determine independent predictors. Further research using standardized methodologies is needed to quantify the relative importance of these risk factors.

**Table 6:** Risk factors identified by researchers, categorized by epidemiological settings.

Setting	Risk factors	References
Human	Immunosuppressive conditions (e.g., HIV+, solid organ transplant, hematologic neoplasms, and primary immunodeficiencies).	Barboni et al. (2008), Astudillo and Bava (2017), and Dumas Marucci et al. (2024)
	Prolonged and persistent diarrhea	Molina et al. (2019)
	Age (> in children under 5 years)	Salomon et al. (2007)
	Residing in vulnerable areas (>poor Water, Sanitation, and Hygiene conditions)	Basualdo et al. (2007), Garbossa et al. (2013), Navone et al. (2006), Rivero et al. (2018), and Rivero et al. (2017a, 2017b)
Animal	Animal management (e.g., housing conditions, feeding practices, and drinking water quality)	Del Coco et al. (2014), Pinto de Almeida Castro et al. (2009), Modini et al. (2016), and Alegre et al. (2024)
	Rainfall and temperature	Hancke and Suárez (2020)
	Type of soil	Tiranti et al. (2011), Del Coco et al. (2014), and Modini et al. (2016)
	Age (> in young animals)	Fontanarrosa et al. (2006), Tiranti et al. (2011), Garro et al. (2016), Pinto de Almeida Castro et al. (2009), Araujo et al. (2011), Modini et al. (2016), and Aguirre et al. (2014)
	Presencia y duration Diarrhea	Garro et al. (2016, 2021), Lombardelli et al. (2019), Pinto de Almeida Castro et al. (2009), and Aguirre et al. (2014)
	Low quality of life index areas	La Sala et al. (2015)
	Overcrowded urban areas	Hancke et al. (2011)
	Contamination of public green spaces	Rubel et al. (2019)
Environmental	Deficient structural conditions and horticultural practices	Falcone et al. (2020, 2023)
	Children and dogs circulating in crops	Falcone et al. (2020, 2023)
	Water contamination indicators (organic matter, turbidity, bacterial pathogens).	Abramovich et al. (2001) and Costamagna et al. (2005)
	Dry season	Cacciabue et al. (2014)

### 3.7 Regional patterns of *Cryptosporidium* spp. co-occurrence

The most common parasitic and bacterial associations reported in each region were also compiled, prioritizing those with statistical significance in the original studies (Table 7).

**Table 7:** Most frequently reported helminths, protozoa, bacteria and viruses reported with *Cryptosporidium* spp. in different regions of Argentina (indicated by a star symbol).

	Regions of Argentina					
	Pampean	Northwestern	Northeastern	Patagonian	GBA	Cuyo
Nonpathogenic protozoa						
*Entamoeba coli*	*		*	*	*	
Pathogenic protozoa						
*Microsporidium (Enterocytozoon bieneusi)*	*					
*Dientamoeba fragilis*	*					
*Cystoisospora belli*	*					
*Giardia lamblia*	*					
*Cyclospora cayetanensis*	*					
*Blastocystis hominis*	*		*	*	*	*
Helmints						
*Strongylides stercoralis*			*			
Bacteria						
*Clostridioides difficile*	*					
*Salmonella*	*					
*Enterocococci*	*					
*E. coli*	*					
Enteropathogenic *E. coli* (EPEC)	*					
Viruses	*					
F + RNA bacteriophages	*					
Group A rotaviruses	*					
Noroviruses	*					
Human polyomaviruses	*					

### 3.8 Laboratory research

Several laboratory research have been employed during the 21st century to elucidate key aspects of *Cryptosporidium* spp. biology, with most studies conducted in the Pampean region during the last decade. Researchers developed an *in vivo* experimental infection model to elucidate the role of apoptosis in disease development (Del Coco et al., 2012). Other studies focused on evaluating vaccine candidates. In one approach, heterologous expression of gp60—a GPI-anchored protein encoded by *C. parvum*—was successfully demonstrated in the ciliate *T. thermophila* (Elguero et al., 2019). Additionally, reverse vaccinology was used to identify novel vaccine candidates against cryptosporidiosis in neonatal bovines (Tomazic et al., 2018). Since GPI-anchored antigens play crucial roles in host cell attachment and invasion, they represent promising targets for vaccination. Using web-based bioinformatic tools, researchers identified the GPI-anchored proteome of *C. parvum*, characterizing 14 putative proteins, including further analysis of CpH1, CpSUB2, and GP60.

Other experimental approaches included comparisons of three stool concentration techniques to improve oocyst recovery (Del Coco et al., 2008), assessments of the effectiveness of various coagulants in eliminating *Cryptosporidium* spp. during water purification (Abramovich et al., 2004), and impact evaluation of the probiotic *Enterococcus faecalis* CECT 7121 on *C. parvum* infection in mice, which yielded promising results (Del Coco et al., 2016).

### 3.9 Interactive map of *Cryptosporidium* spp. studies in Argentina

An interactive web map of the country was created, incorporating study locations along with their geo-references. This map also includes a summary of the data obtained from the three settings (see Supplementary Material) and serves as a tool for quickly visualizing conducted research, author information, and full citations. Link of access: https://scgismaps.maps.arcgis.com/apps/dashboards/09ef2e9537674ce6859ff7a27a53c2f1.

### 3.10 Factors that influence the outcome of the study

In this analysis, several limitations were identified. A significant portion of original data was only reported in conference proceedings or presented as abstracts in scientific meetings. While genetic and experimental approaches are advancing in key areas such as the life cycle, treatment, and diagnosis of these pathogens, many studies were either unpublished as original articles or only partially published, making them ineligible for inclusion in this review. Additionally, most hospital-based patient data were stored in institutional records or government reports without public access. Regarding wild animals, there are reports of *Cryptosporidium* spp. in capybaras, tapirs, and deer, among others. However, all these findings are documented in grey literature, which is not always systematically accessible or peer-reviewed. Many studies collectively assessed risk factors for all intestinal parasites, without specific *Cryptosporidium* spp. analysis. Furthermore, co-infections in different hosts were often unreported, and few studies evaluated the number of detected species or their classification at genus or species level. Another major limitation is the lack of national-level data on *Cryptosporidium* spp. prevalence in humans and animals, which makes it difficult to draw definitive conclusions about the true burden of the parasite across the country. The absence of longitudinal studies further hampers our ability to assess temporal trends in the effectiveness of prevention and control strategies. This lack of long-term data also limits the ability to identify emerging patterns of infection, evaluate changes in transmission dynamics, and assess the impact of public health interventions over time. Additionally, several biases in the data collection methods may influence the reliability of the findings. The non-notifiable disease status of *Cryptosporidium* spp. in Argentina likely leads to underreporting, with many cases going unrecognized or unrecorded in public health databases. This underreporting is especially true for cases occurring in rural and underserved regions, where access to healthcare and diagnostic services may be limited. Furthermore, studies that rely on convenience sampling or cross-sectional designs are prone to selection bias, which may not be representative of the broader population or all potential reservoirs of infection. Despite these challenges, this review provides valuable insights into the incidence of *Cryptosporidium* spp. infections in humans, animals, and its environmental detection across Argentina. It also serves as a valuable guide to address existing gaps and critical aspects of *Cryptosporidium* spp. research, highlighting areas that require further investigation to enhance public health strategies.

## 4 Discussion

This scoping review and analysis of studies conducted in Argentina over the past two decades have highlighted key trends in the epidemiology, diagnosis, and control of *Cryptosporidium* spp. infections in humans, animals, and environmental settings. Our findings indicate that between 2011 and 2020, scientific output on *Cryptosporidium* spp. in Argentina increased steadily, with most studies conducted in the Pampean region of Argentina and reaching its highest level during the 2016–2020 period. This growth likely reflects a combination of increased awareness of the parasite’s public health relevance, advances in diagnostic capabilities, and the incorporation of *Cryptosporidium* spp. into broader One Health and environmental surveillance frameworks. However, a marked decline in the number of publications has been observed in the most recent period (2021–2024). This decrease may be attributable to several factors, including the reallocation of research priorities and funding in response to the COVID-19 pandemic, temporary disruptions to field and laboratory work, and a general shift toward other emerging infectious diseases. These findings highlight the uneven distribution of *Cryptosporidium* spp. research in Argentina, influenced by population density, economic activity, and the availability of institutional support for scientific studies.

The increasing detection of *Cryptosporidium* spp. in both humans and animals across various regions of Argentina aligns with global trends, where the parasite is recognized as a leading cause of water-and food-borne infections as well as an important diarrheal pathogen in humans and animals (Ryan et al., 2014; Dhal et al., 2022). Accordingly, over the years, particularly in the last decade, an increase in publications related to intestinal infections has been observed in Argentina. Indeed, a national review of *Giardia* spp. confirmed they are the most ubiquitous protozoan parasites in the country (Rivero et al., 2020) and highlighted *Cryptosporidium* spp. as another group of pathogens causing diarrhea, which should be analyzed using an integrated approach.

Most epidemiological data on this parasite in Argentina are derived from the Pampean region. This region serves as the country’s agricultural hub and has notable economic and scientific-academic advancements compared to other areas (INDEC, 2019). Indeed, scientific centers are concentrated in this region (Universities, INTA and CONICET) and the best hospitals in the country. The northern regions and Patagonia have increased their contribution to epidemiological studies in recent years, which is directly associated with establishing new centers of scientific research and academic development. However, this disparity in research efforts underscores the need for standardized national surveillance, diagnosis, treatment, and control protocols to ensure more equitable and comprehensive monitoring of *Cryptosporidium* spp. infections.

Identifying specific risk factors in human, animal, and environmental settings, which are inextricably linked, underscores the complexity of *Cryptosporidium* spp. transmission. Our analysis reinforces the need for a *One Health* approach to address the multidimensional nature of this pathogen. Globally, the philosophy that human, animal, and environmental factors must be considered simultaneously to develop effective prevention and control strategies is gaining increasing recognition, and tackling cryptosporidiosis is no exception (Innes et al., 2020). Our analysis of risk factors, in line with those reported in developing countries (Squire et al., 2017; Utaaker et al., 2017; Yang et al., 2021) and others Latin American countries such as Peru, Brazil and Colombia reveals both similarities and critical gaps (Bern et al., 2002; Sánchez et al., 2017; Galvan-Diaz et al., 2020; Higuera et al., 2020; Pacheco et al., 2022). *Cryptosporidium* spp. infections are most prevalent in regions with lower socio-economic conditions, where access to clean water and sanitation is limited. These comparisons underscore the broader regional need for harmonized surveillance strategies across Latin America (Jann et al., 2022). This further reinforces the call for integrated regional efforts to improve diagnostic capacity, reporting systems, and cross-sectoral collaboration in the control of cryptosporidiosis and other neglected protozoan diseases (Jann et al., 2022). The identification of young children as a particularly vulnerable group further emphasizes the need for specific interventions aimed at reducing their risk of infection. Recent global epidemiological studies have highlighted *Cryptosporidium* spp. as the second most important diarrheal pathogens causing life-threatening diarrhea in young children (0–24 months), after rotavirus (Dhal et al., 2022). As stated above, most of the surveys analyzed were focused on humans, particularly children, and the results were mainly based on the epidemiology of intestinal parasite infections in the child population. A recent review of human cryptosporidiosis in the Americas indicates that *Cryptosporidium* spp. is widespread across the continent. In this sense, the average prevalence of *Cryptosporidium* in asymptomatic children in Argentina is similar to the prevalence reported in South America (Galvan-Diaz et al., 2020; Jann et al., 2022). The systematic review and meta-analysis at the continental level found that in developed countries, prevalence is higher when immunological and/or molecular methods are used alongside direct microscopic examination, emphasizing the importance of advanced diagnostic techniques in detecting this pathogen (Jann et al., 2022).

Argentina, while showing scientific advancements in specific regions, particularly the Pampas, still lacks a coordinated national framework. Higher prevalences of *Cryptosporidium* spp. infections were detected in the northern and peri-urban areas of major cities within the Pampean region. These areas suffer from poor sanitation and housing conditions, along with high nutritional vulnerability, all of which create an environment conducive to the transmission and maintenance of parasites. The rapid and unplanned growth of these settlements around large cities fosters conditions that facilitate the spread of infectious diseases. It is important to note that, as with *Giardia* spp. in a previous analysis (Rivero et al., 2020), prevalence reports of *Cryptosporidium* spp. infections may be influenced by the characteristics of the studied populations, which may be more vulnerable due to their socio-economic circumstances. Therefore, it is crucial to report infection data considering these risk factors. This approach would enable the design of more effective control strategies for managing the infection.

*Cryptosporidium* spp. infections are not mandatory to report in Argentina, resulting in a lack of standardized protocols for surveillance, diagnosis, treatment, and control (Ministerio de Salud y Ambiente de Argentina, 2004).

In recent years, diagnostic approaches for detecting *Cryptosporidium* spp. have evolved significantly. Traditional morphological techniques, including concentration methods and staining procedures (e.g., Kinyoun and modified Ziehl-Neelsen), remain widely used due to their low cost and feasibility in routine diagnostics. However, these methods are limited by their low sensitivity and observer-dependent variability (Magi et al., 2006; Luka et al., 2022). Immunological methods, such as enzyme-linked immunosorbent assays (ELISA) and direct fluorescent antibody tests (DFA), offer improved sensitivity and specificity and can detect antigens or oocysts more reliably, although they require access to specialized reagents and infrastructure. Molecular techniques, particularly polymerase chain reaction (PCR)-based assays, represent the most sensitive and specific tools for detecting *Cryptosporidium* species and genotypes, and are crucial for epidemiological surveillance and outbreak investigations (Xiao and Ryan, 2004; Magi et al., 2006; Jann et al., 2022). Nevertheless, the implementation of molecular diagnostics remains limited in public health laboratories across Argentina due to financial and logistical constraints. A critical comparison of these methods underscores the need for national investment in diagnostic capacity building and the integration of molecular tools into routine surveillance systems (Cimino et al., 2015; Luka et al., 2022).

Improving treatment guidelines is also crucial, as Argentina lacks official national protocols for managing cryptosporidiosis besides recommendations on antiparasitic drugs (Nitazoxanide). Additionally, there are important considerations for prescribing these medications to children under two years old, requiring careful analysis of each patient’s health condition and medical history. Fortunately, in recent years, international drug development projects have explored new therapeutic strategies for *Cryptosporidium* spp. (Zhu et al., 2021). Its unique biology challenges traditional drug discovery, necessitating innovative screening methods. Advances in oocyst generation, *in vitro* processing, and continuous 3D cultivation have led to more physiologically relevant assays for identifying inhibitors. These breakthroughs have significantly accelerated the development of anti-*Cryptosporidium* spp. drugs (Zhu et al., 2021; Dhal et al., 2022).

Despite advances in diagnostic methods, such as PCR-based techniques, *Cryptosporidium* spp. remains challenging to detect and treat, particularly in immune-compromised patients. Our understanding of the interactions between *Cryptosporidium* spp., its host, and the factors driving infection and disease, is still incomplete (Pinto and Vinayak, 2021). Additionally, the clinical features of cryptosporidiosis vary not only depending on the species of *Cryptosporidium* but also, in some cases, on the specific subtype involved. In Argentina, there is a need for more comprehensive data on the species of *Cryptosporidium* affecting humans. Further research and improved surveillance will be crucial for enhancing our understanding of the disease and developing more effective control strategies.

Studies have identified four subtypes of *C. hominis* and two of *C. parvum* in Argentina, providing insights into the genetic diversity of the parasite (Peralta et al., 2016; Velásquez et al., 2018). Research conducted between 2010 and 2012 in AIDS patients with chronic diarrhea and cholangiopathy associated with *Cryptosporidium* spp. was the first to reveal details on mixed infections (Velásquez et al., 2012). Evidence has shown that cryptosporidiosis is not limited to HIV-positive patients. There is growing emphasis on studying its impact on immunocompromised individuals, such as organ transplant recipients. This pathogen presents a therapeutic challenge, making it essential to consider *Cryptosporidium* spp. in the differential diagnosis of acute or persistent diarrhea in immunocompromised patients, significantly as the number of transplant patients increases and HIV infections in pediatrics decreases globally (Jung Montanares et al., 2024). Another important area that requires further investigation is the mechanism through which *Cryptosporidium* infects organs outside the digestive system. While the life cycle of the parasite is well understood when it affects the digestive system, little is known about how the infection spreads and impacts other organs (Velásquez et al., 2018). This gap in knowledge presents a crucial opportunity for research to explore the broader pathogenesis of *Cryptosporidium* spp. infections in humans.

Due to the lack of safe drugs or vaccines, *Cryptosporidium* spp. has become a major public health concern. Its oocysts pose a significant environmental challenge, aligning this pathogen closely with the *One Health* framework. The limited number of effective treatment options for both symptomatic and asymptomatic cases highlight a critical gap in public health and veterinary care. In humans, treatment for HIV patients is restricted to combined antiretroviral therapy and azithromycin (Barboni et al., 2008) while in calves, the use of halofuginone shows promise but has limited long-term efficacy (Tomazic et al., 2018). Given these constraints, rigorous, evidence-based preventive measures are essential. In this context, the growing shift from conventional to organic and agroecological farming in Argentina presents an opportunity to promote sustainable development and improve animal welfare. Organic farming emphasizes preventive strategies, such as strict hygiene protocols and water treatment, rather than relying solely on curative approaches (Organización Internacional Agropecuaria, 2019). By adhering to organic production principles, this management model could provide a strong foundation for addressing *Cryptosporidium* spp. infections, highlighting the importance of prevention for human and animal health.

Accordingly, this review focuses on *Cryptosporidium* spp. in animals, identifying key aspects that extend beyond the simple detection of the pathogen in various animal species. These aspects are closely linked to economic and productive processes crucial importance to Argentina. In this context, there is a clear trend towards addressing *Cryptosporidium* spp. and cryptosporidiosis in production animals. For instance, a recent review by De Alba et al. (2023) analyzed cryptosporidiosis in Argentinian calves and demonstrated that *Cryptosporidium* spp. infections are endemic across all the country regions studied (De Alba et al., 2023). Key findings from the country highlight aspects such as the association between subtypes and geographic location, possibly indicating geographic segregation in *C. parvum* subtypes (Del Coco et al., 2014). Additional studies are needed to understand better whether different *Cryptosporidium* spp. subtypes exhibit varying pathogenicity in hosts or if other genetic factors influence the severity of cryptosporidiosis (Del Coco et al., 2014). This underscores the need for further evaluation of *Cryptosporidium* spp.’s impact on animal health and its zoonotic significance (Tiranti et al., 2011; Del Coco et al., 2014; Garro et al., 2021). The pathogen’s low infectious dose, complex life cycle, resistance to environmental conditions, and intricate host interactions establish *Cryptosporidium* spp. as a significant zoonotic pathogen with considerable economic impact. Efforts to effectively inactivate *Cryptosporidium* spp. oocysts, such as composting and anaerobic digestion, are increasing. Additionally, proper animal waste disposal, manure management, and the regular cleaning and renewal of pens are key prevention strategies currently being implemented in dairy farms nationwide (Modini et al., 2016).

Studies about *Cryptosporidium* spp. in pigs are gaining relevance nationwide, with two species detected on farms in various provinces of Argentina (De Felice et al., 2020; Lovera et al., 2022; Alegre et al., 2024). Further surveys should be conducted on farms with different breeding systems and include a wider range of ages/categories to better understand the epidemiology of *Cryptosporidium* spp. infections in Argentina. The results obtained so far show a wider distribution of *C. scrofarum*, which has been detected across all age categories and more frequently than *C. suis*. Both identified *Cryptosporidium* species have zoonotic potential (De Felice et al., 2020), so special measures should be considered to improve the sanitary and safe disposal of pig feces, among other actions, to minimize environmental contamination.

Considering pets, dogs are the most popular companion animals in Argentina. The country ranks highest worldwide in the number of dogs per capita, with a rate twice the global average (Growth-from-knowledge, 2016). *Cryptosporidium* spp. detection in dogs have been reported in five Argentinian provinces across the Northeast, Pampean, and Patagonia regions, encompassing both urban and rural areas. However, these studies focused solely on the presence of oocysts and did not analyze their viability and associated risk factors. Notably, in most cases, dogs were also found to be infected with other parasites of zoonotic concern, reinforcing their role in the transmission and spread of infectious agents. Currently, Argentina lacks national regulations regarding the disposal of dog feces in public spaces. Given this, there is an urgent need to implement public health policies to prevent the environmental spread of dog feces and the parasites they carry. The success of such measures will depend on the development, implementation, and assessment of culturally appropriate public health education strategies (Rivero et al., 2017b).

Assessing the impact of *Cryptosporidium* spp. from a *One Health* perspective would be incomplete without considering the role of synanthropic rodents in their maintenance and transmission (Bidaisee and Macpherson, 2014; Mwangi et al., 2016). Rodents, being abundant and widespread, are major reservoirs of *Cryptosporidium* spp. infections in humans and other animals. Synanthropic rodents host at least 17 species/genotypes of *Cryptosporidium* (Taghipour et al., 2020). Recent local research has highlighted the role of the brown rat (*Rattus norvegicus*) in transmitting protozoan pathogens, providing evidence of co-occurrence between *Cryptosporidium* spp. and *Giardia* spp. (Hancke and Suárez, 2020). Studies have shown that these rodents can harbor *Cryptosporidium* species typically associated with cattle, pigs, other rodents, birds, and mammals (Hancke et al., 2011; Taghipour et al., 2020). Given this, understanding how the composition of *Cryptosporidium* species varies along urban–rural gradients is crucial from an epidemiological standpoint. *Rattus norvegicus*, widely recognized as a valuable model for epidemiological studies, further emphasizes the importance of considering this species as a reservoir for zoonotic endoparasites, which poses a public health risk, particularly in densely populated urban areas (Hancke and Suárez, 2020).

Considering that the occurrence of *Cryptosporidium* spp., in free-ranging and captive wild animals in Argentina is limited, it remains a critical issue that warrants serious consideration. This is particularly important for managing zoo wildlife and addressing potential risks in interface zones where human, domestic animals, and wildlife habitats overlap. The expansion of agricultural and livestock frontiers is transforming forest-rural interface areas into hotspots that demand integrative epidemiological approaches (Schug et al., 2023). Notably, in Patagonia, *Cryptosporidium* spp. were detected in *Mytilus edulis* (Torrecillas et al., 2020), mussels collected by local people for human consumption. This underscores the need for an integrated approach extending to coastal areas. Once again, the collected information reflects that the impact of *Cryptosporidium* spp. can and should be addressed through a broad, comprehensive approach.

Finally, data on the contribution of environmental media to cryptosporidiosis primarily comes from the analysis of surface water samples. In contrast, the absence of oocyst detection in soil samples across all studies highlights the need to improve sedimentation and processing techniques. These improvements should consider the environmental conditions of each region and the types of soil in the surveys to enhance oocyst concentration. The Argentine Food Code (CAA) does not currently mandate the specific monitoring of *Cryptosporidium* presence in drinking water. However, it is important to note that while the CAA does not require specific analysis for *Cryptosporidium*, there are other regulations and guidelines in Argentina that address the surveillance of this protozoan in specific contexts. For example, Resolution 898/2001 from the Ministry of Health approves a guideline that includes procedures for detecting *Cryptosporidium* in water samples, particularly during outbreaks or epidemiological investigations (Ministerio de Salud de la Argentina, 2001). The CAA does regulate certain parameters related to water quality, such as turbidity. Turbidity, which indicates water clarity and the effectiveness of filtration processes, is an important indirect measure, as its reduction helps eliminate particles that may carry pathogens, including *Cryptosporidium* spp. oocysts. Nonetheless, *Cryptosporidium* spp. have been detected even in treated water with low residual turbidity, underscoring the limitations of relying solely on this parameter and reinforcing the need for targeted detection protocols for this parasite (Maciel and Faria Sabogal-Paz, 2016). Notably, no waterborne cryptosporidiosis outbreaks have been reported in the country during the 21st century. However, a recent review of waterborne protozoan parasites in Latin America highlights that surveillance systems for these pathogens are poorly coordinated, have significant limitations, or are entirely absent in many countries in the region (Rosado-García et al., 2017). Consequently, many waterborne protozoan outbreaks likely remain undetected or unreported. In Argentina, incorporating parasite detection into national water quality regulations is essential, particularly given evidence that water classified as safe for human consumption has been found to contain high concentrations of pathogenic protozoan (oo)cysts (Juarez et al., 2015; Rodriguez-Alvarez et al., 2015). In response to this, several research groups advocate for implementing quantitative microbial risk assessment (QMRA) frameworks in public drinking water systems, which would allow for more standardized evaluations based on the origin and characteristics of water sources across different regions (Rodriguez-Alvarez et al., 2015; Prez et al., 2021). These analyses provide a solid foundation for developing effective and context-appropriate diagnostic tools aligned with Argentina’s economic and infrastructural realities.

Altogether, this review highlights the need to reframe *Cryptosporidium* spp. research in Argentina through a coordinated, One Health approach that integrates human, animal, and environmental health. The findings reveal significant knowledge gaps and a fragmented research landscape, which limit our ability to understand the parasite’s transmission dynamics and implement effective control strategies. Public health implications are clear: the lack of nationwide surveillance, limited access to diagnostic tools, and the underreporting associated with *Cryptosporidium*’s non-notifiable status hinder timely detection and response. We recommend the development of a national surveillance system for *Cryptosporidium* spp., incorporating routine diagnostic protocols in both human and veterinary health services. Additionally, enhancing laboratory capacities to perform molecular characterization will help identify circulating species and subtypes, which is critical for understanding zoonotic transmission patterns and targeting interventions.

Longitudinal studies should be prioritized to monitor temporal trends and assess the long-term impact of current prevention and treatment measures, particularly in vulnerable populations and regions with high human-animal-environment interactions, such as rural or peri-urban areas linked to agricultural production. A One Health framework should also guide future research agendas, promoting interdisciplinary collaboration to bridge gaps between medical, veterinary, and environmental sciences. Actionable steps include funding integrated epidemiological studies, supporting open-access databases for shared findings, and developing region-specific guidelines for water safety, animal husbandry, and infection control. By addressing these critical areas, Argentina will be better positioned to mitigate the public health burden of *Cryptosporidium* spp. infections and strengthen its capacity to respond to emerging parasitic threats.

## Data Availability

The raw data supporting the conclusions of this article will be made available by the authors, without undue reservation.
