# Supplementary material for: Cryptosporidium spp. in Argentina: epidemiology and research advances in human, animal, and environmental settings during the 21st century
Source: Front Microbiol. 2025 May 27;16:1592564. doi: 10.3389/fmicb.2025.1592564 (PMC12149211; doi:10.3389/fmicb.2025.1592564)
Supplement: Supplementary file 1 [file Table_1.DOCX]

**Cryptosporidium spp. in Argentina: Epidemiology and Research Advances in Human, Animal, and Environmental Settings During the 21st Century**

Maria Romina Rivero^1*,^ Claudina Vissio^1^, Constanza Feliziani^2^, Carlos De Angelo^3^, Maria Carolina Touz^2^, Karina Tiranti^4^, Joaquín Andrés Lombardelli^4^, Florencia Judith Duartez ^1^, Lumila Curletto ^4^.

*** Correspondence:**rivero.maria@conicet.gov.ar

**Supplementary Table 1.** Animal cryptosporidiosis in Argentina during 2001-2024.

| **Animal Categories** | **Animal species** | **Region of Argentina** | **Province** | **Reference** |
| --- | --- | --- | --- | --- |
| Pets | Dogs | NORTHEAST | Chaco | Enriquez *et al.* 2019 |
| Pets | Dogs | GREAT BUENOS AIRES | Buenos Aires | Fontanarrosa *et al.* 2006 |
| Pets | Dogs | PAMPEAN | Buenos Aires | La Sala *et al.* 2015 |
| Pets | Dogs | PATAGONIAN | Neuquen | Soriano *et al.* 2010 |
| Pets | Dogs | GREAT BUENOS AIRES | Buenos Aires | Rubel *et al.* 2019 |
| Pets | Dogs | NORTWEST | La Rioja | Cerezuela *et al.* 2017 |
| Pets | Dogs | NORTWEST | La Rioja | Cerezuela *et al.* 2017 |
| Pets | Dogs | PATAGONIAN | Rio Negro | Santos *et al.* 2021 |
| Pets | Dogs | PATAGONIAN | Buenos Aires | Venturini *et al.* 2006 |
| Pets | Cat | PAMPEAN | Buenos Aires | Venturini *et al.* 2006 |
| Pets | Geckos *(Eublepharis macularius)* | PAMPEAN | Buenos Aires | Dellarupe *et al.* 2016 |
| Synantropic animals | Brown rat *(Rattus norvegicus)* | PAMPEAN | Buenos Aires | Hancke *et al.* |
| Synantropic animals | Brown rat *(Rattus norvegicus)* | PAMPEAN | Buenos Aires | Hancke *et al.* |
| Farm animals | Calves | PAMPEAN | Córdoba | Tiranti *et al.* 2011 |
| Farm animals | Calves | PAMPEAN | Buenos Aires | Del Coco *et al.* 2018 |
| Farm animals | Calves | PAMPEAN | Buenos Aires | Garro *et al.* 2016 |
| Farm animals | Calves | PAMPEAN | Córdoba | Lombardelli *et al.* 2019 |
| Farm animals | Calves | PAMPEAN | Buenos Aires | Pinto de Almeida Castro *et al.* 2009 |
| Farm animals | Calves | NORTHEAST | Corrientes | Araujo *et al.* 2011 |
| Farm animals | Calves | NORTHEAST | Chaco | Araujo *et al.* 2011 |
| Farm animals | Calves | PAMPEAN | Santa Fe | Modini *et al.* 2011 |
| Farm animals | Calves | NORTHWEST REGION | Salta | Bertoni *et al.* 2021 |
| Farm animals | Calves | NORTHWEST | La Rioja | Cerezuela *et al.* 2017 |
| Farm animals | Calves | PAMPEAN | Buenos Aires | Pezzani *et al.* 2001 |
| Farm animals | Calves | PAMPEAN | Santa Fe | Aguirre |
| Farm animals | Calves | PAMPEAN | Santa Fe | Modini |
| Farm animals | Calves | PAMPEAN | Buenos Aires | Venturini *et al.* 2006 |
| Farm animals | Calves | PAMPEAN | Buenos Aires | Del Coco *et al.* 2014 |
| Farm animals | Calves | PAMPEAN | Buenos Aires | Garro *et al.* 2021 |
| Farm animals | Calves | PAMPEAN | Buenos Aires | Tomazic *et al.* 2013 |
| Farm animals | Calves | PAMPEAN | Santa Fe | Tomazic *et al.* 2013 |
| Farm animals | Calves | PAMPEAN | Córdoba | Tomazic *et al.* 2013 |
| Farm animals | Calves | PAMPEAN | Buenos Aires | Maidana *et al.* 2014 |
| Farm animals | Pigs | PAMPEAN | Buenos Aires | De Felice *et al.* 2020 |
| Farm animals | Pigs | PAMPEAN | Entre rios | De Felice *et al.* 2020 |
| Farm animals | Pigs | NORTHEAST | Misiones | De Felice *et al.* 2020 |
| Farm animals | Pigs | PAMPEAN | Santa Fe | De Felice *et al.* 2020 |
| Farm animals | Pigs | PAMPEAN | Córdoba | De Felice *et al.* 2020 |
| Farm animals | Pigs | PAMPEAN | La Pampa | De Felice *et al.* 2020 |
| Farm animals | Pigs | PAMPEAN | Córdoba | Lovera *et al.* 2022 |
| Farm animals | Pigs | NORTHEAST | Corrientes | Alegre *et al.* 2024 |
| Farm animals | Horses | PAMPEAN | Buenos Aires | Venturini *et al.* 2006 |
| Farm animals | Goat | PAMPEAN | Buenos Aires | Venturini *et al.* 2006 |
| Farm animals | Sheep | PAMPEAN | Buenos Aires | Venturini *et al.* 2006 |
| Wild animals | Coypus *(Myocastor coypus)* | PAMPEAN | Buenos Aires | Martino *et al.* 2012 |
| Wild Animals | *Mussels (Mytilus edulis)* | PATAGONIAN | Chubut | Torrecillas *et al.* 2020 |
| Wild Animals | *Mussels (Mytilus edulis)* | PATAGONIAN | Chubut | Torrecillas *et al.* 2020 |
| Wild animals in captivity | Puma *(Puma concolor)* | PAMPEAN | Buenos Aires | Pezzani *et al.* 2001 |
| Wild animals in captivity | Black howler monkey (Alouatta caraya) | PAMPEAN | Buenos Aires | Servian *et al.* 2020 |
| Wild animals in captivity | Black spider monkey *(Ateles sp.)* | PAMPEAN | Buenos Aires | Venturini *et al.* 2006 |
| Wild animals in captivity | Chimpanzee *(Pan troglodytes)* | PAMPEAN | Buenos Aires | Venturini *et al.* 2006 |
| Wild animals in captivity | Baboons *(Papio sp.)* | PAMPEAN | Buenos Aires | Venturini *et al.* 2006 |
| Wild animals in captivity | Tufted capuchin *(Sapajus apella)* | PAMPEAN | Buenos Aires | Venturini *et al.* 2006 |
| Wild animals in captivity | Black howler monkey (Alouatta caraya) | PAMPEAN | Buenos Aires | Venturini *et al.* 2006 |
| Wild animals in captivity | Guinea pigs *(Cavia porcellus)* | PAMPEAN | Buenos Aires | Venturini *et al.* 2006 |
